# Multidisciplinary Analysis of Differences Between Finisher and Non-finisher Ultra-Endurance Mountain Athletes

**DOI:** 10.3389/fphys.2019.01507

**Published:** 2019-12-10

**Authors:** Pedro Belinchón-deMiguel, José Francisco Tornero-Aguilera, Athanasios A. Dalamitros, Pantelis T. Nikolaidis, Thomas Rosemann, Beat Knechtle, Vicente Javier Clemente-Suárez

**Affiliations:** ^1^Departamento de Enfermería, Facultad de Siencias Biomédicas y de la Salud, Universidad Europea de Madrid, Madrid, Spain; ^2^Facultad de Ciencas del Deporte, Universidad Europea de Madrid, Madrid, Spain; ^3^Department of Physical Education & Sport Sciences, Aristotle University of Thessaloniki, Thessaloniki, Greece; ^4^School of Health and Caring Sciences, University of West Attica, Egaleo, Greece; ^5^Exercise Physiology Laboratory, Nikaia, Greece; ^6^Institute of Primary Care, University of Zurich, Zurich, Switzerland; ^7^Gesundheitszentrum St. Gallen, St. Gallen, Switzerland; ^8^Grupo de Investigación en Cultura, Educación y Sociedad, Universidad de la Costa, Barranquilla, Colombia

**Keywords:** psychology, odontology, nutrition, training, stress, mountain running

## Abstract

Ultra-endurance races are one of the most physically and psychologically demanding sports, depending performance on several elements. The aims of the present study were (i) to analyze differences in selected psychophysiological parameters between finisher and non-finisher ultra-endurance mountain athletes, and (ii) to analyze modifications in psychophysiological parameters before and after an ultra-endurance mountain event. Selected psychophysiological variables were assessed in 46 finishers and 24 non-finishers in two over 100 km ultra-endurance races were examined. We found how an ultra-endurance mountain race produced dehydration, a decrease in systolic blood pressure, weight and leg strength muscle values, as well as an increase in heart rate and rate of perceived exertion values. Finishers presented lower systolic blood pressure, weight, body mass index, half marathon time and fluid intake before competition day compared to non-finishers. In addition, body mass index, pre-race hydration, and performance in lower distance races were predictors of performance in these ultra-endurance mountain races.

## Introduction

Ultra-endurance mountain events are increasing their popularity and the number of participants, showing an exponential increase in recent years (Knechtle et al., 2009b). Human performance under extreme conditions has been understudied mainly due to the sample-accessibility difficulties. Previous research has focused on the evaluation of anthropometric characteristics demonstrating that low body fat percentage is a key success factor in ultra-endurance events (Barandun et al., 2012), as well as describing acute physiological effects showing increased protein catabolism and muscle breakdown (Jamart et al., 2012), and autonomic sympathetic modulation (Valenzano et al., 2016).

These extreme events can also produce increased creatinine kinase and urea concentrations, due to muscle destruction and catabolic status of athletes (Marklund et al., 2013), blood lactate values related to the anaerobic threshold (Clemente-Suarez, 2015), sustained hemoglobin levels due to increased erythropoiesis in order to compensate for exercise-induced hemolysis (Schumacher et al., 2002). In addition, increased triglyceride metabolism (Volek et al., 2016), overexpression of the sympathetic nervous system (Belinchon-deMiguel and Clemente-Suárez, 2018) and cardiovascular effort which could reach up to 71% of the maximum heart rate (Valenzano et al., 2016) have also been reported. From a psychological perspective, athletes have a significant anxiogenic response, expressed by the levels of post-exercise cortisol values (Nicolas et al., 2011), fact also related with the metabolic response in these extreme events.

Other studies analyzed the role of the number of training sessions (Zaryski and Smith, 2005), the effect of chronological age (Clemente-Suarez and Nikolaidis, 2017), the contribution of different biomechanical and morphological factors (Hoffman and Fogard, 2011) as well as emotional and personality constructs (Lane and Wilson, 2011), as key performance parameters for ultra-endurance events. Latest studies observed greater performance in ultra-endurance sport events with an appropriated hydration and optimal nutritional status (Peters, 2003; Costa et al., 2014), oral health status and periodontal disease (Needleman et al., 2013, 2015; Ashley et al., 2015; Frese et al., 2015), higher training intensity and volume (Knechtle et al., 2012), higher pain tolerance (Schutz et al., 2012; Freund et al., 2013) and stress management (Baker et al., 2005; O’Neil and Steyn, 2007). Moreover, a previous study analyzed specific psychological parameters such as the vital commitment, coping with anxiety, self-perception, stress perception and psychological flexibility showing their relationship with athletic performance (Hughes et al., 2003).

Moreover, ultra-endurance athletes are accustomed to consuming non-steroidal anti-inflammatory drugs (NSAIDs) or central nervous system activators, as caffeine, which can lead to gastrointestinal discomfort, as well as potential side effects on the gastrointestinal tract and kidneys (Joslin et al., 2013); however most of the athletes are not aware of the side effects, while a widely use among ultra-endurance athletes has been reported despite the medical and professional advise (Wichardt et al., 2011). Furthermore, it has been postulated that most of the ultra-endurance athletes do not adjust their fluid and energy balance requirements during the race (Belinchon-deMiguel and Clemente-Suárez, 2018), mostly due to a lack of knowledge and supervision. Nevertheless, successful athletes have shown an appropriate liquid and fuel ingestion, prior and during the race, fulfilling their demands and keeping an appropriate water and energy balance (Clemente-Suarez, 2015).

Ultra-endurance races are a complex multifactorial phenomenon, affected by different physiological and psychological parameters. Then, to better understand the psychophysiological variables related to performance in this events, as well as, the effect of this extreme races in the organism we conducted the present research with the aims of (i) to analyze differences in selected psychophysiological parameters between finishers and non-finishers ultra-endurance mountain athletes, and, (ii) to analyze modifications in psychophysiological parameters before and after an ultra-endurance mountain event. The initial hypothesis were (i) finisher ultra-endurance mountain athletes would present a different psychophysiological profile than non-finishers, and (ii) ultra-endurance mountain race would decrease weight and strength of participants as well as increase cardiovascular response and deshydration status.

## Materials and Methods

### Participants

Seventy volunteer (46 finishers and 24 non-finishers) male ultra-endurance mountain athletes were analyzed (chronological age 42.7 ± 7.8 years; height 173.2 ± 6.8 cm; body mass 69.2 ± 7.2 kg; and body mass index 23.1 ± 1.5 kg m^2^) of a total of 146 races participants. Prior to the start of the study, the experimental procedures were explained to all the participants, who gave their voluntary written informed consent in accordance with the Declaration of Helsinki. The study was approved by the European University of Madrid Bioethics Committee (CIPI/002/17).

### Ultraendurance Race

The Canfranc-Canfranc ultra-endurance mountain race edition 2016, composed of 100 km of distance and 8848 m of positive change of altitude and 17696 accumulated changes of altitude, as well as and the GTP (Gran Trail de Peñalara) ultra-endurance mountain race edition 2016 with 112 km and 5100 m of positive change of altitude and 10200 m of accumulated change of altitude were analyzed.

#### Materials and Method

The following parameters were analyzed in the ultra-endurance athletes following procedures of previous authors in ultraendurance events (Suarez et al., 2011; Belinchon-deMiguel and Clemente-Suárez, 2018; Belinchón-Demiguel and Clemente-Suárez, 2019).

#### Physiological Measurements

• Body mass was measured by using a SECA scale model 714 with a precision of 100 g (range: 0.1–130 kg), located on a flat and smooth surface and calibrated at zero. Participants were barefoot and with minimal clothes. Once located in the center of the platform, they remained without their body being in contact with surrounding objects, with the weight evenly distributed on both feet facing forward.
• Body height was measured with a height rod incorporated in the scale SECA model 714 with a precision of 0.1 mm (range: 60–200 cm). Participants stood up barefoot with the head oriented in the Frankfort plane that joins the inner edge of the eye socket and the upper one of the external auditory meatus, arms on both sides of the trunk, extended and with palms touching external face of the thighs, heels together touching the lower end of the vertical surface with the inner edge of the feet in the 45 to 60°, occipital area, scapular, buttocks, posterior face of the knees and calves touching the vertical surface of the anthropometer.
• Body Mass Index (BMI = Weight in kg/Size^2^ in meters) was calculated according to the World Health Organization.
• Heart Rate (HR) was recorded with a Polar V800 HR monitor (Kempele, Finland). HR was analyzed before and immediately after the race. In order to record baseline values, athletes remained in the waiting room without any disturb.
• The Omron M6 Confort (Osaka, Japan) was used for the blood pressure measurement (BP) with the participants seated with their arm flexed at the level of the heart in a waiting room without any disturb.
• Blood oxygen saturation (BOS) was calculated using an oximeter OXYM4000 (Quirumed, Madrid), placed in the index finger of the right arm.
• The isometric hand-grip strength was assessed using a TKK 5402 dynamometer (Takei Scientific Instruments, Co., Ltd.). Each athlete’s hand grip strength was measured in the dominant hand. Athletes were placed sitting with 0 degrees of shoulder flexion, 90 degrees of elbow flexion and the forearm in neutral. The highest result of the two trials was recorded.
• Lower limbs strength was evaluated by a horizontal jump test. Athletes were standing behind a line marked on the ground with feet slightly apart. A two-feet take-off and landing was used, with swinging of the arms and bending of the knees to provide forward drive. Athletes had 3 attempts, analyzing the highest result; the measurement was taken from take-off line to the nearest point of contact on the landing, which is the back of the shoe heels.
• Hydration status was assessed by a urine measurement using the urine color chart methodology (Armstrong et al., 1994).
• Forced vital capacity (FVC) and forced expiratory volume (Fev1) were calculated using a QM-SP100 (Quirumed, Spain) spirometer in a maximum inhale-exhale cycle.
• Rate of perceived exertion (RPE) was measured using the 6–20 scale (Costa et al., 2014).

Moreover, training, nutritional, psychological and odontological differences between finisher and non-finisher ultra-endurance mountain runners were analyzed by a questionnaire filled out the week before the race reporting the following parameters.

#### Training Parameters

T otal distance (km), positive or negative change of altitude accumulated race (m), training experience (years), mountain race experience (years), marathon best mark (min), half marathon time (min), expected race position, expected time (min), weeks injured, training per week (h), training per session (min), training sessions per week, mean speed training per week (min/km), positive change of altitude accumulated per week (m), change of altitude accumulated per week (m), resistance training sessions (per week), load percentage during resistance training (% of 1RM), stretching time per week (min) and stretching sessions per week.

#### Nutritional Parameters

Carbohydrates meals during the pre-competition week (n°) and fluid intake before competition day (Knechtle et al., 2009b).

#### Psychological Parameters

Perceived stress (as measured by the Perceived Stress Scale (PSS-14) (Remor, 2006), and general mental health status (as measured by the Acceptance and Action Questionnaire, AAQ-II (Bond et al., 2011).

#### Odontological Parameters

Grinding or clenching teeth while performing usual tasks, nibbles objects, bites his nails, wakes up with fatigue in mouth muscle, consumption of vitamin C pills, wet pillow upon awakening and repeated burps.

### Statistical Analysis

To analyze the data, we used the SPSS statistical package (version 22.0; SPSS, Inc., Chicago, IL, United States). Normality of the sample was determined with the Shapiro–Wilk event. To analyze differences between pre and post samples, a dependent-*t*-test was performed for all the variables as they presented a parametric distribution. To analyze differences between finishers and non-finishers an independent-*t*-test was conducted. The effect size was evaluated by the Cohen d event. Finally a correlation analysis between study variables and finalization of the probe was calculated by the Pearson Test. For all comparisons, the significance index of *p* < 0.05 was accepted.

## Results

Results are reported as mean ± *SD* values. Regarding pre-race physiological differences between finishers and non-finishers, systolic blood pressure and body weight presented significant differences, being greater in non-finishers. Greater isometric hand grip strength and explosive strength of the lower limbs, measured through the horizontal jump length, were greater in non-finishers, as well as the FVC and RPE values (Table 1).

**TABLE 1:** Differences between mountain ultra-endurance finishers and non-finishers in pre-race body mass and psychophysiological parameters.

							**95% Interval confidence**	
**Variable**	**Finisher**	**Non-finisher**	***T***	***P***	**Effect size**	**% Change**	**Lower**	**Higher**
Body mass (kg)	69.27.2	75.47.1	–3.557	0.001	–0.86	–4.0	1.75	2.63
RPE	8.41.7	9.22.0	–1.707	0.092	5.54	113.5	–10.52	–8.81
Urine colorimetry	2.21.4	2.51.2	–0.808	0.422	1.39	91.2	–3.11	–1.51
IHS (N)	46.26.5	50.59.5	–1.794	0.080	0.22	3.1	–5.23	0.57
Temperature °C	36.60.4	36.50.5	0.632	0.529	–0.09	–0.1	–0.38	0.35
Horizontal jump length (cm)	125.769.9	132.973.8	–0.416	0.679	–0.53	–29.5	10.03	32.08
Systolic blood pressure (mmHg)	122.813.2	129.011.7	–2.053	0.044	–1.30	–14.0	11.97	21.9
Diastolic blood pressure (mmHg)	71.39.3	74.212.9	–1.097	0.276	–0.21	–2.7	–2.14	4.65
HR (bmp)	65.011.1	67.29.8	–0.854	0.396	2.10	36.0	–30.17	–21.87
BOS (%)	96.91.2	96.61.5	1.059	0.293	–0.27	–0.4	–0.13	1.16
FVC	2016.32143.4	2428.92576.7	–0.736	0.464	0.20	21.3	–233.79	266.32
FEV1	582.071.8	589.790.5	–0.321	0.750	–0.81	–87.7	–7.89	104.38

*IHS, isometric hand strength; RPE, rating of perceived effort; HR, heart rate; BOS, blood oxygen saturation; FVC, forced vital capacity; Fev1, forced expiratory volume.*

Significant differences were found between finishers and non-finishers, regarding the half marathon time, in where finishers had significant lower time. In addition, non-finishers presented a greater number of injured weeks, a greater number of training sessions, as well as a lower mean speed during training and a lower percentage of load during resistance training, compared with finishers (Table 2).

**TABLE 2:** Differences between mountain ultra-endurance finishers and non-finishers in training and psychological parameters.

						**95% Interval confidence**	
**Variable**	**Finisher**	**Non-finisher**	***T***	***P***	**Effect size**	**Lower**	**Higher**
**Training parameters**							
Distance (km)	106.34.8	106.44.8	–0.106	0.916	0.02	–2.21	2.46
Positive change of altitude accumulated race (m)	6485.11829.1	6438.51828.8	0.106	0.916	–0.03	–920.49	827.38
Negative change of altitude accumulated race (m)	6485.11829.1	6438.51828.8	0.106	0.916	–0.03	–920.49	827.38
Years sport practice	24.012.0	24.212.7	–0.038	0.970	0.02	–6.25	6.50
Years of mountain race practice	6.33.8	5.43.7	–0.091	0.728	0.03	–1.86	2.05
Marathon best mark (min)	227.867.2	230.480.8	–0.128	0.898	0.04	–37.84	43.02
Half marathon time (min)	89.88.2	101.030.4	–2.171	0.034	1.37	0.877	21.52
Expected race position	67.869.6	68.172.4	–0.014	0.989	0.00	–40.85	41.43
Expected time (min)	1314.8229.6	1295.5273.0	0.314	0.754	–0.08	–142.43	103.67
Weeks injured	2.13.4	3.16.5	–0.872	0.386	0.29	–1.34	3.42
Training per week (h)	10.45.0	11.24.7	–0.707	0.482	0.16	–1.58	3.32
Training per session (min)	97.335.6	99.041.2	–0.181	0.857	0.05	–17.24	20.69
Training sessions per week	4.71.1	5.11.6	–1.203	0.233	0.36	–0.26	1.07
Medium speed training per week (min/km)	6.01.8	5.51.4	1.124	0.266	–0.28	–1.48	0.41
Positive change of altitude accumulated per week (m)	1792.11083.8	1714.21605.9	0.222	0.825	–0.07	–780.93	625.29
Change of altitude accumulated per week (m)	3700.02066.2	3523.83210.1	0.256	0.799	–0.09	–1552.25	1199.87
Resistance training (0 no. 1 yes)	0.50.5	0.50.5	–0.616	0.540	0.00	–0.17	0.33
% of load in resistance training (% of 1RM)	59.721.8	44.225.4	1.844	0.075	–0.71	–32.67	1.66
Stretch (0 no. 1 yes)	0.90.1	1.00.0	–0.775	0.441	1.00	–0.036	0.08
Stretching per week (min)	13.35.7	12.24.9	0.799	0.427	–0.19	–3.87	1.66
Stretching sessions per week	4.31.4	4.21.4	0.155	0.877	–0.07	–0.80	0.68
**Physiological parameters**							
Perceived stress (PPS)	28.45.7	29.64.3	–0.959	0.341	0.21	–1.35	3.86
General mental health (AAQII)	15.06.6	14.18.2	0.475	0.636	–0.14	–4.43	2.72

Regarding questionnaire analysis between finisher and non-finishers, significant differences were found in BMI and height, presenting finishers lower values. Non-finishers presented lower chronological age, lower fluid intake before competition day and lower grinding or clenching the teeth while performing usual tasks (Table 3).

**TABLE 3:** Differences between mountain ultra-endurance finishers and non-finishers in anthropometrical, nutritional and odontological parameters.

						**95% Interval confidence**	
**Variable**	**Finisher**	**Non-finisher**	***T***	***P***	**Effect size**	**Lower**	**Higher**
**Anthropometrical parameters**							
Age (years)	42.77.8	40.86.7	1.067	0.289	–0.24	–5.46	1.65
Body mass index (kg/m^2^)	23.11.5	24.12.0	2.439	0.017	0.67	0.19	1.86
Height (cm)	173.26.8	176.75.7	–2.305	0.024	0.51	0.48	6.65
**Nutritional parameters**							
Carbohydrates meals the competition week (n°)	6.02.7	6.01.6	–0.128	0.899	0.00	–1.12	1.28
Fluid intake before competition day (l)	2.41.0	3.32.6	–2.105	0.039	0.90	0.05	1.87
**Odontological parameters**							
Grinding or clenching your teeth while performing your usual tasks (0 no. 1 yes)	0.10.3	0.30.4	–1.916	0.046	0.67	–0.01	0.40
Nibbles objects	0.20.4	0.20.4	0.145	0.885	0.00	–0.24	0.21
Bites his nails	0.30.4	0.30.4	–0.368	0.714	0.00	–0.20	0.30
He wakes up with fatigue muscle pain (0 no. 1 yes)	0.10.3	0.00.2	0.622	0.536	–0.14	–0.23	0.12
Vitamin C pills	0.00.3	0.00.2	0.721	0.474	–0.17	–0.19	0.09
Wet pillow upon awakening	0.10.3	0.00.2	0.978	0.332	–0.24	–0.23	0.08
Repeated burps	0.00.2	0.00.2	0.058	0.954	0.00	–0.11769	0.11104

During the post ultra-endurance race event, a significant increase was presented in the urine colorimetry, RPE and HR, while significant decreases were observed in the explosive strength of the lower limbs, systolic blood pressure and body weight (Table 4).

**TABLE 4:** Modification of study variables before and after the ultraendurance events.

**Variables**	**Pre**	**Post**	***T***	***P***	**Effect size**	**Higher**
Body mass (kg)	68.67.0	66.56.3	10.031	0.000	–0.31	2.63
RPE	8.21.6	17.91.7	–22.927	0.000	5.74	–8.81
Urine colorimetry	1.91.1	4.22.1	–5.893	0.000	1.95	–1.52
IHS (N)	45.46.2	47.87.7	–1.667	0.110	0.37	0.58
Horizontal jump length (cm)	112.171.9	91.159.5	3.890	0.000	–0.29	32.09
Systolic blood pressure (mmHg)	123.112.9	106.112.0	6.893	0.000	–1.31	21.93
Diastolic blood pressure (mmHg)	70.88.8	69.59.9	0.748	0.459	–0.14	4.66
Temperature (°C)	36.60.4	36.60.9	–0.058	0.954	0.02	0.36
Heart rate (bpm)	63.010.5	89.010.5	–12.697	0.000	2.47	–21.89
Blood oxygen saturation (%)	97.11.1	96.61.8	1.603	0.117	–0.44	1.16
FVC	2287.02239.0	2270.72199.1	0.132	0.896	–0.01	266.33
FEV1	572.080.1	523.785.8	1.799	0.088	–0.60	104.39

*RPE, rating of perceived effort; IHS, isometric hand grip strength; HR, FVC, forced vital capacity; Fev1, forced expiratory volume.*

Finally, we found a negative significant correlation between systolic blood pressure (*r*: −0.237; *p*: 0.044), height (*r*: −0.262; *p*: 0.024), weight (*r*: −0.389; *p*: 0.001), half marathon time (*r*: −272; *p*: 0.034), fluid intake before competition day (*r*: −0.253; *p*: 0.039) and the finalization of the ultraendurance mountain probe.

## Discussion

The aims of the present research were (i) to analyze differences in selected psychophysiological parameters between finisher and non-finisher ultra-endurance mountain athletes, and (ii) to analyze modifications in psychophysiological parameters before and after an ultra-endurance mountain event. The data presented here showed that finishers and non-finishers showed differences in psychophysiological, training, anthropometrical, nutritional, and odontological variables. In addition, it was shown that finishing an ultramarathon mountain event produced dehydration, a significant increase in rating of perceived effort and heart rate, and a significant decrease in weight, horizontal jump length, and systolic blood pressure.

Regarding pre-race differences between finishers and non-finishers, half marathon time presented significant and large ES lower values in finisher than non-finishers, result consequent with other studies where lower marathon time was also correlated to greater performance in ultra-endurance race events above 100 km (Knechtle et al., 2010). This results emphasized the capacity to perform higher intensity as a parameter related with ultraendurance performance, high intensity allow ultraendurance athletes to improve their aerobic metabolic system in the same way or even higher than traditional low intensity and high volume training (Clemente-Suárez et al., 2014; Clemente-Suárez and Ramos-Campo, 2019). Furthermore, another performance predictor according to the literature is body composition, where lower BMI values have been linked with greater performance in long-distance ultra-endurance events (Knechtle, 2014), consequent with our data since finishers presented lower BMI values and weight and height presented a negative correlation with the finallization of the probe. In addition, traditionally, greater body height has been linked with greater performance in this type of events, especially in female ultra-endurance events (Whyte et al., 2000). However, considering body height as an isolated value may be inappropriate, since there are other anthropometric and biomechanical factors related to this parameter, such as the circumference of the calves or upper arm, that need to be taken into account (Knechtle et al., 2009a). This is in accordance with our data, since body height was greater in non-finishers.

Regarding the water intake the day before the race event, finishers presented a smaller consumption of liquid fluid compared with non-finishers. Interestingly, previous authors have highlighted the importance of appropriate fluid intake during the competition instead of greater fluid intake before the competition as it can lead to a deterioration of performance (Von Duvillard et al., 2004). Thus, incorrect hydration can lead to a hyper-hydration state that can conclude to hyponatremia as a consequence of electrolyte imbalance, producing alterations in the cardiovascular response and alterations in athlete performance, either to a dehydration process in where the health and performance of the athlete are also going to be compromised (Von Duvillard et al., 2004). In addition, previous authors have reported that experienced athletes have greater self-perception and self-knowledge according to their hydration needs; hence, they arrive at the race event with optimal hydration levels, avoiding dehydration or hyponatremia (Hoffman et al., 2019). This fact may partially explain hydration and dehydration results, since finishers presented greater experience in this type of race events compared to non-finishers, as finishers presented lower pre-race hydration levels, same colorimetry as non-finishers before competition, but significant lower colorimetry after the race event. Furthermore, previous authors have showed how values over 4 in the colorimetry chart, and acute weight loss over the 2% lead to not only a loss of performance, but to health-related risks (Tornero-Aguilera and Clemente-Suarez, 2019). Our data showed a weight loss of 3.2% in both groups, even though it seems inevitable to avoid dehydration in events as demanding as ultra-endurance mountain events, it can be established as a health and performance key factor.

Physiological values, such as systolic blood pressure, presented lower values in finishers compared to non-finishers. In this line, the correlational analysis showed how the systolic blood pressure presented a negative significant correlation with the finalization of the probe. Lower systolic blood pressure was previously reported as a physiological adaptation to aerobic training, highlighting these training adaptations due to endurance training in these extreme ultraendurance events (Portier et al., 2001). Yet, there was a significant decrease in the systolic blood pressure after the race event, as in previous researches concerning a 51.2 km ultra-sustainability race event (Belinchon-deMiguel and Clemente-Suárez, 2018). Regarding the cardiovascular response, it remained at the aerobic threshold level, with a mean HR increase of 41% from baseline values.

Another acute effect of this type of events is a decrease in muscle strength values, not generalized, since only the strength of the lower limbs was negatively affected, which is consequent with previous studies in similar ultra-endurance events (Belinchon-deMiguel et al., 2019), explained due to the higher implication of lower body muscles. However, significant increases were found in isometric hand grip strength, which leads us to think that the greater sympathetic activation which occurs in this type of events (Belinchon-deMiguel and Clemente-Suárez, 2018), can lead to increases in muscle strength, as it has been shown in other stressful and extreme conditions, such a military contexts (Tornero-Aguilera et al., 2017; Tornero-Aguilera and Clemente-Suarez, 2018). This tendency was not similar with an ultra-endurance Paralympic athlete, where hand strength values decreased after an ultra-endurance mountain event, probably due to the fact the upper body muscles were the principal implicated in this event (Belinchon-deMiguel et al., 2019).

The nutritional analysis did not present any differences according to the intake of carbohydrate meals during the race week between finishers and non-finishers; however, it is known that correct storage of energy is essential for adequate performance, since in ultra-endurance mountain events the energy balance is extremely negative (Clemente-Suarez, 2015). Nerveless, correct hydration (by urine colorimetry) showed a large ES between finisher and non-finisher, fact that reinforce previous author in the importance of hydration in ultraendurance events (Costa et al., 2014). Correct nutrition is a key factor in ultra-endurance events, but it should be analyzed in greater depth in order to determine its scope for the successful completion during mountain ultra-endurance events.

The psychological analysis revealed no differences between finisher and non-finisher athletes regarding perceived stress or mental health in general. One possible explanation is that participants in this ultraendurance events reported low levels of stress and good general mental health compared to the general population (Bond et al., 2011). In this line, the rated of perceived exertion increased significantly at the end of the ultra-endurance event, a fact that is in line with previous investigations that showed RPE values of 17.5 ± 2.1 with a distance greater than 90 km (Belinchón-Demiguel and Clemente-Suárez, 2019). Finally, in the odontological parameters analyzed we found no differences between finishers and non-finishers, result opposite to previous studies that found relationships between poor oral health and sport performance (Needleman et al., 2015). The participants of this study presented correct odontological health patterns, fact that would explain the lack of significant differences between the group studied. However, we must consider that the general odontological health presented by both athlete’s groups were good, a fact that could explain the lack of differences between finishers and non-finishers.

### Limitation of the Study

The principal limitations of the present study were the lack of biochemical control of muscle destruction (urea, ck), autonomic modulation, and stress hormones (cortisol, alpha amylase) for a better understanding of a multivariable phenomenon that is the ultra-endurance events. With a larger study sample, predictive models of performance in this type of extreme events could be designed.

## Conclusion

An ultra-endurance mountain race produced dehydration, a decrease in systolic blood pressure, weight and leg strength muscle values, as well as an increase in heart rate and RPE values. Finishers presented lower systolic blood pressure, weight, body mass index, half marathon time and fluid intake before competition day compared to non-finishers. In addition, body mass index, pre-race hydration, and performance in lower distance races were predictors of performance in these ultra-endurance mountain races.

This information could help both, trainers and athletes, to improve training, nutritional and psychological interventions as well as, to make more safe the participation in this extreme events.

## Data Availability Statement

The datasets generated for this study are available on request to the corresponding author.

## Ethics Statement

The studies involving human participants were reviewed and approved by the University Bioethics Committee (CIPI/002/17). The patients/participants provided their written informed consent to participate in this study.

## Author Contributions

PB-D and VC-S collected the data. PB-D, JT-A, and VC-S analyzed the data. P-BD, JT-A, AD, PN, TR, BK, and VC-S wrote the manuscript.

## Conflict of Interest

The authors declare that the research was conducted in the absence of any commercial or financial relationships that could be construed as a potential conflict of interest.
